# Re-vegetation Improves Soil Quality by Decreasing Soil Conductivity and Altering Soil Microbial Communities: A Case Study of an Opencast Coal Mine in the Helan Mountains

**DOI:** 10.3389/fmicb.2022.833711

**Published:** 2022-03-30

**Authors:** Zihao Li, Bingru Liu, Zifeng Chen, Dachuan Mao, Xingsheng Jiang

**Affiliations:** ^1^Key Laboratory of Ecological Protection of Agro-pastoral Ecotones in the Yellow River Basin, National Ethnic Affairs Commission of the People’s Republic of China, North Minzu University, Yinchuan, China; ^2^Ningxia Key Laboratory for the Development and Application of Microbial Resources in Extreme Environments, North Minzu University, Yinchuan, China; ^3^China Coal Research Institute (CCRI), Beijing, China; ^4^Ningxia Forestry and Grassland Administration, Yinchuan, China

**Keywords:** opencast coal mine, re-vegetation, bacterial community, fungal community, Helan mountains

## Abstract

Microbial communities constitute a diverse genetic resource pool in the soil and are key indicators of soil health and quality. How re-vegetation affects soil microbial diversity and community composition at the dump of an opencast coal mine is largely unknown. Using high-throughput sequencing, we performed a comparative study of the bacterial and fungal communities from non-vegetated (bare land) soil and from areas re-vegetated by *Astragalus laxmannii*, *Halogeton arachnoideus*, and *Artemisia desertorum* at an opencast coal mine in the Helan Mountains in western China. These results indicated that re-vegetation significantly reduced soil conductivity. The soils re-vegetated by all three plant species showed greater richness of bacterial species than the bare land, and soils re-vegetated with *A. desertorum* and *A. laxmannii* showed significantly greater richness of fungal species than bare land. The bacterial and fungal β-diversity values differed significantly between vegetated and non-vegetated soil, and these differences were more pronounced for bacterial communities than for fungal communities. Re-vegetation significantly increased the relative abundances of *Proteobacteria* and *Bacteroidota* and decreased the relative abundance of *Chloroflexi*. The decreasing soil conductivity that occurred with re-vegetation was found to be an important environmental determinant of the soil microbial community. This study provides evidence that re-vegetation may enhance soil quality via decreasing soil conductivity and altering the soil microbial community, and *A. laxmannii* was found to be a more effective species than *H. arachnoideus* or *A. desertorum* with respect to decreasing soil conductivity and altering the soil microbial communities in the Opencast Coal Mine arid region. This work may provide a helpful guideline for selection of plant species for re-vegetation projects.

## Introduction

As one of the most important energy resources worldwide, coal has affected our daily life for nearly three centuries. However, coal mining has caused a series of environmental problems, as opencast coal mining alters the landscape, induces geological disasters, destroys vegetation, and hence reduces ecosystem functions (Liao, 2009; Wu et al., 2014). Resolution of these environmental problems is a global imperative. Topographical reconstruction (Verma et al., 2011; Gong et al., 2021), soil reconstruction (Dimitriu et al., 2010; Lei et al., 2016), and re-vegetation (Neldner and Ngugi, 2017; Hou et al., 2019) are currently the main methods used for ecological restoration. Studies have shown that topographical reshaping can affect rainfall infiltration and regulate water erosion processes, and hence retain water in the soil and promote vegetation restoration (Zheng et al., 2010; Lin et al., 2015). Utilization of biochar for soil reconstruction can improve dump soil water content and increase plant stem height, leaf area, and aboveground biomass (Huang et al., 2021). However, in addition to topographical reconstruction and soil reconstruction, re-vegetation is crucial for the ecological restoration of mines and is the premise of ecosystem recovery.

A promising new method, re-vegetation after topographical or soil reconstruction, is becoming increasingly popular in dump soil remediation due to its potential for restoring biodiversity and reconstructing ecosystem functions (Hou et al., 2019). However, suitable plant species are needed for re-vegetation in mine dump soils, as the plant community should induce ecosystem succession and eventually restore ecosystem functions (Srivastava et al., 2012; Li P. et al., 2019). Studies have shown that a longer re-vegetation time range is associated with higher soil quality compared to use of bare land (James et al., 1996). Studies have also shown that re-vegetation plays an important role in mitigating the negative effects of surface runoff, wind erosion, and dust pollution on the local environment (Peng, 2000). In the meantime, plant root exudates and plant litter can increase soil fertility and recruit diverse microbial communities that improve energy and nutrient cycling of dump soils (Massaccesi et al., 2015; Zeng et al., 2017). Currently, drought-tolerant trees such as *Pinus tabuliformis* and *Robinia pseudoacacia*, shrubs such as *Artemisia ordosica* and *Hippophae rhamnoides*, and herbs such as *Medicago sativa*, *Melilotus officinalis*, *Agropyron cristatum*, and *Setaria viridis*, are commonly used as reclamation species in northwest China (Zhao et al., 2013; Yuan et al., 2016, 2018; Guo et al., 2020). Studies have found that legumes, Compositae, and gramineous plants are effective pioneers in opencast mine ecological restoration. In previous studies, soil physiochemical properties such as pH (Liu et al., 2017), C:N (Shrestha and Lal, 2011), and conductivity (Miller et al., 2012) have been commonly used as ecological indicators of restoration. However, in the early recovery stages of degraded soils, soil physiochemical properties are limited regarding their ability to reflect the effects of ecological restoration (Davidson and Janssens, 2006), while the microbial community is more sensitive than soil physiochemical properties to environmental changes and thus can be a better indicator of ecological restoration (Dimitriu et al., 2010; Dangi et al., 2012). The microbial community plays an essential role in ecosystem functioning, e.g., in carbon and nitrogen fixation and organic matter decomposition (van der Heijden et al., 2008; Chaparro et al., 2012). Soil microorganisms are closely related to aboveground plant species, and distinct microbial communities are often observed with various plant species used for re-vegetation (Deng et al., 2020). The re-vegetated plants influence the soil microbial community via litter decomposition and root exudates, while microbes may increase plant growth by retaining water and offering available nutrients (Wang et al., 2011; Zhang et al., 2013b; Massaccesi et al., 2015; Yang et al., 2017; Zeng et al., 2017). Therefore, microbial diversity and community composition can be good indicators of re-vegetation status. It remains largely unknown how the soil microbial community changes with the vegetation during ecological remediation in mining areas.

The Helan Mountains are rich in coal resources. Presently, the exploitation of coal resources at the Helan Mountains is mainly done through opencast mining that has seriously damaged surface vegetation. The irregular dumping of coal mine waste has had a huge impact on the landscape, water environment, and local biodiversity, resulting in opencast coal mining in the area being discontinued. The ecological restoration of the dump soil continues to be a major problem. Therefore, the local government has banned mining and has initiated re-vegetation of the dump soil using the plant species *Halogeton arachnoideus*, *Artemisia desertorum*, and *Astragalus laxmannii.* Seeds of the three plant species were sown in the dump site. Two years later, soils were sampled in re-vegetated and non-re-vegetated quadrats. The objectives of this study were: (1) to explore whether re-vegetation would increase microbial (bacterial plus fungal) diversity; (2) to examine the microbial community composition among re-vegetated plant species; and (3) identify the key environmental factors driving variation in the microbial community composition during re-vegetation.

## Materials and Methods

### Site Description

The study site was located at Dafeng Mine (106°1’–106°13’E, 39°1’–39°9’N), the Helan Mountains, Ningxia, China. It was once an important coal production base in the north-central part of the Helan Mountains, an area adjacent to Gulaben of the Autonomous Region (Inner Mongolia), and has a distribution area of about 200 km^2^ (Chen et al., 2005). The mean annual precipitation is 195.3 mm, which mostly falls in the summer. The mean annual average temperature is 8.61°C. The soil types that have not been damaged by coal mining are mainly sierozems (Qu et al., 2018). The soil at the site is in a low nutrient condition and is very thin. The dump soil is composed of coal gangue, slag, and coal mining waste combined with a small amount of sierozem from native topographic soil stripped prior to mining.

**FIGURE 1:**
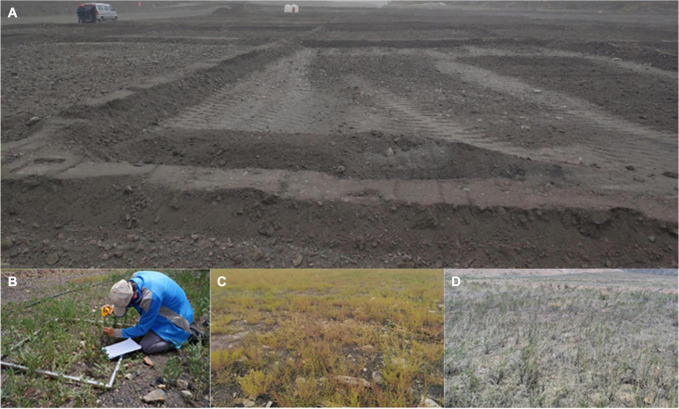
Photographs illustrating quadrats of bare land that were re-vegetated by different plant species. The images were taken by Zihao Li and Dachuan Mao. **(A)** Land before re-vegetation (2018); **(B–D)** after re-vegetation with *A. laxmannii*, *H. arachnoideus*, and *A. desertorum*, respectively (2020).

The native vegetation is dominated by *Stipa breviflora* and *Agropyron cristatum* and few small shrubs. Most of the lands here are degraded and have almost no plant growth due to coal mining activities. The artificially re-vegetated plant species are *Halogeton arachnoideus*, *Artemisia desertorum*, and *Astragalus laxmannii*. In June 2018, seeds of each plant species were sown at the dump site in 16 30 m × 30 m quadrats, with four replicates for each plant species. The intervals between any adjacent quadrats were at least 30 m to achieve randomization (Figure 1). Bare land without seeds sown was treated as the control treatment of non-vegetation. All plants were grown under semi-arid conditions without irrigation, fertilization, nor perturbation after planting. Field sampling was conducted during the growing season (August 2020). Within each quadrat of each plant species, a 1 m × 1 m sub-quadrat was established for soil sampling and plant community investment. In each quadrat, five random soil cores were collected and pooled as a composite sample. In total, 16 samples were collected for three plant species and the bare land. Soil was transported to the lab on ice, sieved (2 mm), and divided into two parts. One part was air dried for soil property measurements and the other part was stored at −80°C for molecular analysis. The basic information of each quadrat is listed in Table 1.

**TABLE 1:** Information for plant communities in each quadrat.

Plant species	Life-form	Total coverage (%)	Plant height (cm)	Density (number/m^2^)	Aboveground biomass (g/m^2^)
*A. laxmannii*	Perennial herbs	68.33 ± 10.27	28.33 ± 0.58	26 ± 8	277 ± 42
*H. arachnoideus*	Annual herbs	66.67 ± 12.47	49.5 ± 6.24	37 ± 15	191.67 ± 39.31
*A. desertorum*	Semishrub herb	48.33 ± 6.23	14.89 ± 8.09	26 ± 12	277 ± 84.45

### Measuring Plant and Soil Properties

For aboveground biomass measurement, the aboveground parts of plants in each quadrat were harvested and dried at 65°C to a constant weight. Soil samples were air dried, and soil physiochemical parameters were measured as described previously (Xu et al., 2021). Briefly, soil pH and electrical conductivity (EC) were measured in a soil water suspension (1:5, w/v) using the DDS-307A (Leici, Shanghai, PRC). The soil water content (SWC) was measured gravimetrically. Total N (TN) was determined by the semimicro-Kjeldahl method. Total P (TP) was determined by the Mo–Sb colorimetric method, and total potassium (TK) was determined by means of flame atomic emission spectrophotometry. The available N in the soil was measured by the alkaline hydrolysis diffusion method; available P (AP) was extracted with a NaHCO_3_ solution, and its content was determined by the Mo–Sb anti-spectrophotometric method. Available potassium (AK) was extracted with an NH_4_OAc solution and measured by flame atomic emission spectrophotometry, and soil organic carbon (SOC) was measured by the K_2_Cr_2_O_7_ titration method (Liu, 2018).

### Molecular Analyses

Soil metagenomic DNA was extracted using the E.Z.N.A. ^®^ (Omega Bio-tek, Norcross, GA, United States) DNA extraction kit according to the manufacturer’s instructions. Then, the V3-V4 hypervariable regions of the bacterial 16S rRNA gene were amplified using a polymerase chain reaction (PCR) amplifier (2720, ABI, United States), with the primer set 338F (5′-ACTCCTACGGGAGGCAGCAG-3′) and 806R (5′-GGACTACHVGGGTWTCTAAT-3′), while the ITS1 region of fungal sequences was amplified using the primer set ITS1F (5′-CTTGGTCATTTAGAGGAAGTAA-3′) and ITS2R (5′-GCTGCGTTCTTCATC GATGC-3′).

The PCR reactions were performed in a 20 μL mixture and comprised 4 μL 5 × *TransStart FastPfu* buffer, 2 μL 2.5 mM dNTPs, μL forward primer (5 μM), 0.8 μL reverse primer (5 μM), 0.4 μL *TransStart FastPfu* DNA polymerase, 1 μL template DNA, and 11 μL ddH_2_O. Triplicate PCR reactions were conducted for each sample. For bacterial sequences, the thermal program for amplification was 3 min of initial denaturation at 95°C followed by 35 cycles of 30 s at 95°C, 30 s at 55°C, 30 s at 72°C, and a final extension of 10 min at 72°C. For fungal sequences, the thermal program for amplification was 3 min of initial denaturation at 95°C, 27 cycles of 30 s at 95°C, 30 s at 55°C, 30 s at 72°C, and a final extension of 10 min at 72°C. The amplicon sizes were determined and selected by running 2% agarose gel electrophoresis in 1.0 × Tris-acetate-EDTA (TAE) buffer, and the amplicons were purified using the AxyPrep DNA Gel Extraction Kit (AP-GX-250, Axygen, United States). The purified amplicons were quantified on a microplate reader (FLX800, BioTek, United States) with a Quant-iT PicoGreen dsDNA Assay Kit (P7589, Invitrogen, United States) and pooled. Samples were normalized in equimolar amounts in the final mixture. DNA library construction was performed on an Illumina platform following the manufacturer’s instructions. Finally, target DNA was sequenced on a Miseq PE300 platform. Sequencing was conducted by MajorBio (Shanghai, PRC). Analyses were conducted in triplicate for each sample. Original sequence data were deposited at the National Center for Biotechnology Information (NCBI), Sequence Read Archive (SRA) with the accession number of PRJNA787760.

### Bioinformatics

Quality control of original sequences was processed using fastp (Chen et al., 2018)^1^ software. Paired-end reads were merged using the FLASH software (Magoè and Salzberg, 2011). Chimera checking was conducted by USEARCH 8 in the *de novo* mode using UPARSE (Edgar, 2013)^2^. Operational taxonomic units (OTUs) were obtained by clustering the sequences at an identified threshold of 97%. The taxonomic information of each sequence was assigned using the ribosomal database project (RDP) classifier^3^. Bacterial sequences were blasted against the Silva 16S rRNA (v138) database, while fungal sequences were blasted against the Unite (Release 8.0) database. The threshold value of blast was 70% (Stackebrandt and Goebel, 1994). All samples of sequences were resampled to 29,524 and 34,654 sequences per sample of bacterial and fungal communities, respectively, referring to the sample with the lowest sequence read for downstream analyses.

### Statistical Analyses

Differences in soil physiochemical properties and bacterial and fungal diversity indices among plant species were tested by one-way analysis of variance (ANOVA) using SPSS 19.0 (IBM, United States). Differences in relative abundances of the bacterial and fungal phyla among plant species were shown by bar plots in OrginLab 2018. All other statistical analyses were performed using R (4.0.4). The shared and unique OTUs among different sites (Venn diagram) were calculated using the *VennDiagram* package. The differences between bacterial and fungal β-diversity among plant species and the correlation analysis of microbial community and environmental factors were checked using the ‘vegan’ package (v2.5.7). Non-metric multidimensional scaling (NMDS) and analysis of similarities (ANOSIM) were complementarily used to analyze the differences. Redundancy analysis (RDA) and canonical correspondence analysis (CCA) were used to check the relationships between bacterial and fungal β-diversity (differences in community composition) and environmental variables.

## Results

### Variation of Soil Physiochemical Properties Among Bare Land and Different Re-vegetated Plant Species

Soil water content during sampling was extremely low, ranging from 1.78% to 3.26% (Table 2). Dump soil pH ranged from 7.9 to 8.75, representing slightly alkaline conditions (Table 2). The dump soils re-vegetated by *A. laxmannii* showed higher pH values (8.75) than soils re-vegetated by other plant species and bare land. Compared with the bare land, re-vegetation significantly decreased soil electrical conductivity. Soils re-vegetated by *A. desertorum* showed higher soil conductivity values (44.03 us/cm) than soils re-vegetated by other plant species. No differences in soil organic carbon, total P, available P, or total potassium were observed between soils from bare land and those from different re-vegetated plant species. Re-vegetation significantly increased available potassium content over that of bare land. Soils re-vegetated by *H. arachnoideus* showed higher available potassium content (231.75 mg/kg) than other re-vegetated soils. In addition, soils re-vegetated by *A. laxmannii* had higher total N contents (1.05 g/kg) than bare land or other re-vegetated soils (Table 2).

**TABLE 2:** Soil physiochemical properties among bare land and different re-vegetated plant species.

	SOC (g/kg)	TN (g/kg)	TP (g/kg)	AP (mg/kg)	TK (g/kg)	AK (mg/kg)	pH	EC (us/cm)	SWC (%)
Bare land	42.44 ± 14.27*a*	0.98 ± 0.25*ab*	0.47 ± 0.04*a*	4.17 ± 0.56*a*	95.29 ± 4.38*a*	102.22 ± 10.93*b*	7.92 ± 0.13*b*	76.08 ± 21.74*a*	2.76 ± 0.45*b*
*A. laxmannii*	44.89 ± 22.42*a*	1.05 ± 0.28*a*	0.56 ± 0.2*a*	3.96 ± 1.39*a*	92.85 ± 11.59*a*	115.99 ± 20.17*b*	8.75 ± 0.33*a*	15.74 ± 3.13*c*	3.26 ± 0.21*a*
*H. arachnoideus*	39.91 ± 5.99*a*	0.65 ± 0.13*c*	0.41 ± 0.02*a*	4.46 ± 1.14*a*	88.71 ± 4.59*a*	231.75 ± 23.39*a*	8.18 ± 0.3*b*	19.63 ± 2.96*c*	1.78 ± 0.16*c*
*A. desertorum*	37.08 ± 5.63*a*	0.7 ± 0.05*bc*	0.48 ± 0.02*a*	4.21 ± 0.48*a*	84.36 ± 4.61*a*	123.03 ± 7.6*b*	7.95 ± 0.19*b*	44.03 ± 12.83*b*	2.65 ± 0.37*b*

*AK/AP, available K/P; EC, electrical conductivity; SOC, soil organic C; SWC, soil water content.; TK/TN/TP, total K/N/P. Different lower-case letters indicate significant differences at P < 0.05.*

### Effects of Re-vegetation on the Soil Microbial Community

#### Effects of Re-vegetation on Soil Microbial Diversity

**FIGURE 2:**
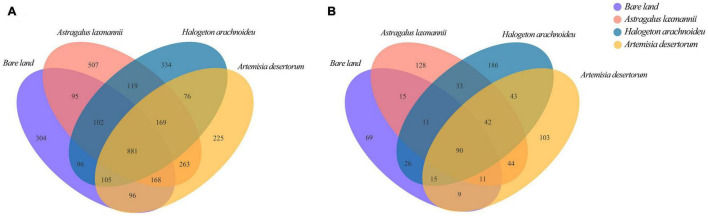
Venn diagram of **(A)** bacterial and **(B)** fungal communities in soils from bare land and re-vegetated areas.

For all 16 samples, we obtained a total of 29,524 bacterial sequences that were clustered into 3,540 OTUs. We also obtained 34,654 fungal sequences that were clustered into 825 OTUs. Re-vegetated and bare land soils shared 881 bacterial OTUs. *A. laxmannii*, *H. arachnoideus*, and *A. desertorum* re-vegetated soils had 95, 96, and 96 bacterial OTUs relative to bare land, respectively (Figure 2A), while they were 15, 26, and 9 for fungal OTUs (Figure 2B). *H. arachnoideus* re-vegetated soils shared more fungal OTUs relative to bare land than those re-vegetated with other plant species (Figure 2). In addition, bare land, *A. laxmannii*, *H. arachnoideus*, and *A. desertorum* soil bacteria had 304, 507, 334, and 225 unique OTUs, respectively (Figure 2A), while soil fungi had 69, 128, 186, and 103 unique OTUs (Figure 2B). Re-vegetated soils had more unique fungal OTUs than bare land soil (Figure 2).

**FIGURE 3:**
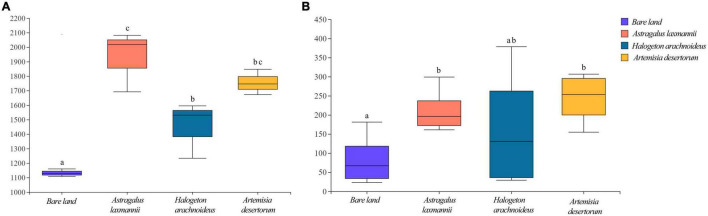
Richness of **(A)** bacterial and **(B)** fungal communities in soils from bare land and those re-vegetated with different plant species. Different lower-case letters indicate significant differences based on least significant difference tests.

Compared to bare land, re-vegetated soils showed significantly higher bacterial richness, being higher in soils re-vegetated by *A. laxmannii* and lower in soils re-vegetated by *H. arachnoideus* (Figure 3A). Soils re-vegetated by *A. laxmannii* and *A. desertorum* showed significantly higher fungal richness than that of bare land (Figure 3B).

**FIGURE 4:**
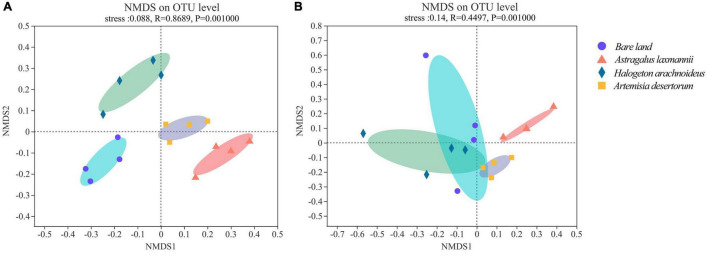
Non-metric multidimensional scaling (NMDS) of **(A)** bacterial and **(B)** fungal communities in soils from bare land and re-vegetated areas with different plant species. OTU, operational taxonomic unit.

The stress indices of NMDS analyses were less than 0.20 for both bacterial and fungal models, suggesting a reliable model behavior. In bacterial NMDS plots, the samples showed different clusters regarding bare land and re-vegetated plant species (Figure 4A), while samples of bare land and *H. arachnoideus* showed overlap in fungal NMDS plots (Figure 4B). These results could also be supported by the ANOSIM results, where bacterial β-diversity was significantly different between bare land and re-vegetated soils (Table 3). Similar results were observed for the fungal community, but there was no significant difference in β-diversity between bare land and *H. arachnoideus* (Table 3). In addition, the differences in bacterial community composition were stronger than those of fungal community composition (Table 3). The above results suggest that re-vegetation had a greater influence on bacterial than on fungal diversity, both in species richness and compositional differences among sites.

**TABLE 3:** Analyses of similarity (ANOSIM) for bacterial and fungal communities among bare land and different re-vegetated plant species based on abundance considering Bray-Curtis dissimilarity matrices.

Comparisons among re-vegetation plant species	Bacteria	Fungi
	*r* ^a^	*r* ^a^
Bare land vs. *A. laxmannii*	1.000*	0.458*
Bare land vs. *H. arachnoideus*	0.813*	0.042
Bare land vs. *A. desertorum*	0.969*	0.344*
*A. laxmannii* vs. *H. arachnoideus*	1.000*	0.698*
*A. laxmannii* vs. *A. desertorum*	0.813*	0.958*
*H. arachnoideus* vs. *A. desertorum*	0.792*	0.510*

^a^Significance level, *p < 0.05.

#### Effects of Re-vegetation on Soil Microbial Community Composition

**FIGURE 5:**
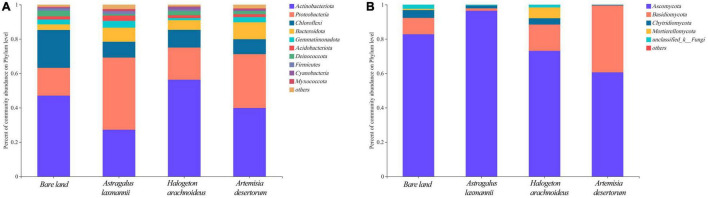
Horizontal abundance characteristics of **(A)** bacterial and **(B)** fungal communities in soils from bare land and re-vegetated areas with different plant species.

**FIGURE 6:**
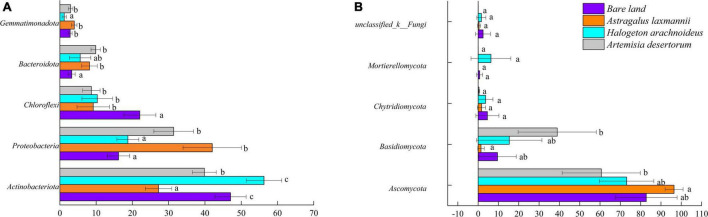
Relative abundances of **(A)** dominant bacterial and **(B)** fungal phyla in soils from bare land and re-vegetated areas with different plant species. Different lower-case letters indicate significant differences based on least significant difference tests.

To further describe how re-vegetation affected soil microbial community composition, the differences in relative abundances of abundant bacterial and fungal genera among re-vegetation treatment were analyzed. The bacterial OTUs were affiliated with 35 phyla, and the top five most abundant genera were *Actinobacteria* (42.59%), *Proteobacteria* (27.08%), *Chloroflexi* (12.56%), *Bacteroidota* (6.73%), and *Gemmatimonadota* (2.74%, Figure 5). The mean relative abundances of the above five phyla all differed among soils from re-vegetated and bare land (Figure 6A). Most interestingly, the relative abundance of *Chloroflexi* was lower in re-vegetated soil than in bare land, while those of *Proteobacteria* and *Bacteroidota* were higher in *A. laxmannii* and *A. desertorum* soils than in bare land soils (Figure 6A).

For the fungal community, the OTUs comprised 10 genera. The mean relative abundances of *Ascomycota* (78.23%), *Basidiomycota* (16.30%), *Chytridiomycota* (2.57%), and *Mortierellomycota* (1.76%) were higher than 1%, and only the relative abundances of *Ascomycota* and *Basidiomycota* differed among soils re-vegetated by different plants and bare land (Figures 5B, 6).

#### Key Factors Mediating Variation of Soil Microbial Communities Among Re-vegetated and Non-vegetated Soils

**FIGURE 7:**
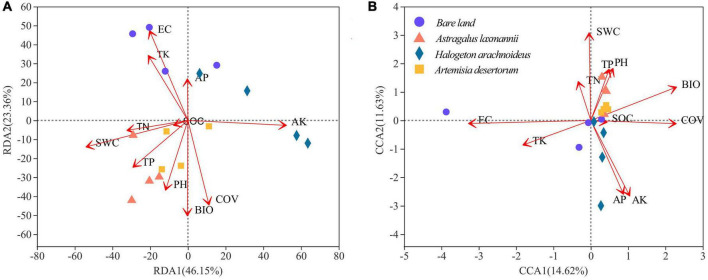
Redundancy analysis (RDA) of **(A)** bacterial and canonical correspondence analysis (CCA) of **(B)** fungal communities based on environmental factors. AK/AP, available K/P; BIO, biomass; COV, coverage; EC, electrical conductivity; SOC, soil organic C; SWC, soil water content; TK/TN/TP, total K/N/P.

The first and second axes of the RDA explained 46.15% and 23.36%, respectively, of the variation in the total bacterial community (Figure 7A); the respective values for the fungal community (CCA) were 14.62 and 11.63% (Figure 7B). This indicated that bacterial rather than fungal communities were more influenced by environmental factors. Our results showed that soil conductivity (*R*^2^ = 0.6318, *P* = 0.003), available potassium (*R*^2^ = 0.6086, *P* = 0.005), and total potassium (*R*^2^ = 0.3771, *P* = 0.041) had significant effects on the variation among bacterial communities. In addition, soil conductivity (*R*^2^ = 0.5441, *P* = 0.008), and available potassium (*R*^2^ = 0.3919, *P* = 0.032) also showed significant effects on fungal community composition.

## Discussion

### Re-vegetation Decreased Soil Conductivity

Soil physiochemical properties are key indicators of soil quality and are important for the growth of plants and microbes. Previous studies have shown that re-vegetation can increase soil quality as indicated by available N, C, and P, as well as by decreased soil salinity (Walker et al., 2004; He et al., 2014; Deng et al., 2019). High salinity indicates high soil conductivity. Thus, our results were in accordance with those previous studies which found that re-vegetation decreased soil salinity. Two factors may account for such a phenomenon. First, plant metabolism can increase land cover and reduce surface water evaporation, inhibiting salt from rising to the surface through capillary action in the soil (Li X. et al., 2019; Liu et al., 2019). Second, plants can absorb salt through their root systems (Islam et al., 2022). Soil re-vegetated with *H. arachnoideus* and *A. laxmannii* showed lower conductivity than that re-vegetated with *A. desertorum*. This is probably because *H. arachnoideus* and *A. laxmannii* are halophyte species, which can absorb higher amounts of salt from the soil through their root systems (Wang and Zhang, 2011; Li, 2017).

In addition, soils re-vegetated by *A. laxmannii* showed higher soil nitrogen content. This could be due to the root nodules of *A. laxmanni*, whose commensal diazotrophs may provide a central source for increasing soil N content, as the nutrients in such soils are fixed in mineral forms, leading to low availability for plants (Hailu Gunnabo et al., 2021). Nevertheless, our results provide evidence that re-vegetation increases soil quality by decreasing soil conductivity. Merely relying on the reconstruction of vegetation cannot guarantee the successful recovery of ecosystem functioning (Orozco-Aceves et al., 2017). The soil microbial community is important in mediating ecosystem functioning via biogeochemical processes and hence is vital for ecosystem stability (Schulz et al., 2013).

### Re-vegetation Alters the Soil Microbial Community via Decreasing Conductivity

Bacterial community composition was significantly different between re-vegetated and non-vegetated soils and among soils re-vegetated by different plant species. This is not surprising, because vegetation types and plant species are reported to influence soil microbial diversity and community composition (Šourková et al., 2005; Li et al., 2013). Our results suggested that re-vegetation altered the soil microbial community, and this was in line with a previous re-vegetation study at a dump site. Bacterial richness was higher in re-vegetated than in bare land soils, while fungal richness was not. In addition, the ANOSIM results also suggested that bacterial community composition in bare land was significantly different from that of re-vegetated soils, while fungal communities in soils from bare land were similar to those in *H. arachnoideus* soils. Furthermore, greater percentages of variance explained by environmental parameters were observed for bacterial communities than for fungal communities (Figure 7). All of the above results can be explained by bacteria and fungi having different responses to re-vegetation (Elliott et al., 2015; Ren et al., 2018). Fungi usually have larger body size than bacterial and are more tolerant to environmental changes (de Vries et al., 2018).

Organic matters secreted by root exudates and nitrogen fixed by rhizobia in the node of Legumes can provide more energy for microbial growth and reproduction, resulting in higher microbial diversity. These provide positive feedback on improving plant growth and soil quality (Hu et al., 2018; Zhalnina et al., 2018; Schulte et al., 2021). Thus, the greater soil bacterial richness of *A. laxmannii* re-vegetated soils over those other plant species may be explained by *A. laxmannii*’s well-developed root system, which *H. arachnoideus* does not have (Bell et al., 2014). In addition, *A. laxmannii*, which is a legume, may increase soil microbial richness via nitrogen fixation. Moreover, bacterial community composition in soils re-vegetated by *A. laxmannii* differed more than in *H. arachnoideus* and *A. desertorum* soils (Table 3). These results indicated that *A. laxmannii* should be a better choice of plant species than *H. arachnoideus* or *A. desertorum* for application to dump soil re-vegetation engineering. This was in line with a previous study (Zhang et al., 2013a). Our results showed that *Proteobacteria*, *Actinobacteria*, *Chloroflexi*, *Bacteroidota*, and *Gemmatimonadota* were the main bacterial taxa among soils re-vegetated by different plant species, and for fungal taxa, these were *Ascomycota*, *Basidiomycota*, and *Chytridiomycota.* The relative abundance of *Actinobacteria* was not strictly high or low between re-vegetated and non-vegetated soils, but it was the dominant bacterial taxon (Figure 5). *Actinobacteria* has hyphae and is reported to be oligotrophic and stress-tolerant, being able to survive in extremely arid and bare soils (Srinivasan et al., 1991). Similar functions have been found in *Ascomycota* (Egidi et al., 2019). *Actinobacteria* and *Ascomycota* were, respectively, dominant in bacterial and fungal communities (Figure 5). The relative abundance of *Chloroflexi* was lower in re-vegetated soils, while the relative abundances of *Proteobacteria* and *Bacteroidota* were higher in *A. laxmannii* and *A. desertorum* re-vegetated soils (Figure 6). This is probably because *Chloroflexi* are oligotrophs (Srinivasan et al., 1991; Egidi et al., 2019; Xian et al., 2020) that typically prefer barren habitats, while *Proteobacteria* and *Bacteroidota* are copiotrophs (Goldfarb et al., 2011; Bennett et al., 2020) that prefer nutrient-rich environments. This could be an indicator that re-vegetation has altered the soil environment, compared to bare land.

Previous studies have shown that in various habitats and at various spatial/time scales, soil microbial communities are mediated by several key factors, e.g., soil pH (Xu et al., 2021), soil available nutrients (Zhao et al., 2019), and soil organic C (Liu et al., 2015). Aside from these factors, our results showed that in coal mine dump soils, re-vegetation inducing decreased conductivity was the key driver of the soil microbial community. This is not surprising, since soil conductivity is associated with soil salinity (Rhoades et al., 1989). Except for halophilic bacteria, the activity and biomass of most bacteria decrease significantly in high salinity environments (Martin et al., 1999) due to high permeation pressure.

In addition to soil factors, microbes are also influenced by aboveground plant species. Different plant species may offer various types of litter for microbes and different root exudates via the root-soil-microbial interface (Yao et al., 2014). Differences in plant biomass and coverage may also influence microclimate and habitat complexity (Waldrop et al., 2006). However, fungi are usually more closely related to plant species than bacteria, while plant coverage and biomass were not significantly correlated with fungal community composition. To explain this, further study at a longer time scale is needed (de la Fuente et al., 2021).

## Conclusion

Soil microbial diversity and community composition are important ecological indicators of a soil system. This study suggested that re-vegetation decreased soil salinity and changed soil bacterial diversity and community composition more than those of fungal taxa. The soils re-vegetated by all three plant species showed higher bacterial richness than that of bare land, while only *A. desertorum* and *A. laxmannii* re-vegetated soils showed significantly higher fungal richness compared to bare land. The most abundant bacterial and fungal taxa were *Actinobacteria* and *Ascomycota*, respectively. Re-vegetation significantly decreased the relative abundance of *Chloroflexi*, and re-vegetation with *A. desertorum* and *A. laxmannii* increased the relative abundances of *Proteobacteria* and *Bacteroidota.* The decreasing soil conductivity with re-vegetation was found to be the key factor driving changes in both bacterial and fungal communities. Our research provides significant guidance for future re-vegetation engineering at dump sites in this area and could be of great significance to improving soil quality by decreasing soil conductivity and increasing microbial diversity. We also found that *A. laxmannii* is a better choice than *H. arachnoideus* or *A. desertorum* for such projects.

## Data Availability Statement

The datasets presented in this study can be found in online repositories. The names of the repository/repositories and accession number(s) can be found below: https://www.ncbi.nlm.nih.gov/genbank/, PRJNA787760.

## Author Contributions

BL first conceptualized the study and reviewed and revised the first draft in subsequent work. ZL, DM, and XJ collected data and samples in the field. ZL, ZC, and DM analyzed the data. ZL wrote the manuscript. ZC provided linguistic assistance. All authors contributed to the article and approved the submitted version.

## Conflict of Interest

The authors declare that the research was conducted in the absence of any commercial or financial relationships that could be construed as a potential conflict of interest.

## Publisher’s Note

All claims expressed in this article are solely those of the authors and do not necessarily represent those of their affiliated organizations, or those of the publisher, the editors and the reviewers. Any product that may be evaluated in this article, or claim that may be made by its manufacturer, is not guaranteed or endorsed by the publisher.

## Footnotes

1 https://github.com/OpenGene/fastp, version 0.20.0

2 http://drive5.com/uparse/, version 7.1

3 http://rdp.cme.msu.edu/, version 2.2

## References

[B1] Bell C. Carrillo Y. Boot C. M. Rocca J. D. Pendall E. Wallenstein M. (2014). Rhizosphere stoichiometry: are C: N: P ratios of plants, soils, and enzymes conserved at the plant species-level? *New Phytol.* 201 505–517. 10.1111/nph.12531 24117992

[B2] Bennett A. C. Murugapiran S. K. Hamilton T. L. (2020). Temperature impacts community structure and function of phototrophic Chloroflexi and Cyanobacteria in two alkaline hot springs in Yellowstone National Park. *Environ. Microbiol. Rep.* 12 503–513. 10.1111/1758-2229.12863 32613733 PMC7540483

[B3] Chaparro J. M. Sheflin A. M. Manter D. K. Vivanco J. M. (2012). Manipulating the soil microbiome to increase soil health and plant fertility. *Biol. Fertil. Soils* 48 489–499. 10.1007/s00374-012-0691-694

[B4] Chen S. Zhou Y. Chen Y. Gu J. (2018). Fastp: an ultra-fast all-in-one FASTQ preprocessor. *Bioinformatics* 34 i884–i890. 10.1093/bioinformatics/bty560 30423086 PMC6129281

[B5] Chen Y. Li J. Yang B. Zhang S. (2005). Monitoring coal fires based on remotely sensed data and GIS technique in coalfields: a case study of Rujigou coal field in Ningxia. *China. J. China Univ. Min. Technol.* 34 226–230. 10.3321/j.issn:1000-1964.2005.02.021 30704229

[B6] Dangi S. R. Stahl P. D. Wick A. F. Ingram L. J. Buyer J. S. (2012). Soil microbial community recovery in reclaimed soils on a surface coal mine site. *Soil Sci. Soc. Am. J.* 76 915–924. 10.2136/sssaj2011.0288

[B7] Davidson E. A. Janssens I. A. (2006). Temperature sensitivity of soil carbon decomposition and feedbacks to climate change. *Nature* 440 165–173. 10.1038/nature04514 16525463

[B8] de la Fuente J. I. Martínez-González C. R. Oros-Ortega I. Guevara G. Bandala V. M. Córdova-Lara I. (2021). *Melanogaster coccolobae* sp. nov. (Paxillaceae, Boletales), a tropical hypogeous fungus from the urban areas of Quintana Roo, Mexico. *Acta Bot. Mex.* 128:e1896. 10.21829/abm128.2021.1896

[B9] de Vries F. T. Griffiths R. I. Bailey M. Craig H. Girlanda M. Gweon H. S. (2018). Soil bacterial networks are less stable under drought than fungal networks. *Nat. Commun.* 9:3033. 10.1038/s41467-018-05516-5517 PMC6072794 30072764

[B10] Deng J. Bai X. Zhou Y. Zhu W. Yin Y. (2020). Variations of soil microbial communities accompanied by different vegetation restoration in an open-cut iron mining area. *Sci. Total Environ.* 704:135243. 10.1016/j.scitotenv.2019.135243 31787305

[B11] Deng J. Yin Y. Luo J. Zhu W. Zhou Y. (2019). Different revegetation types alter soil physical-chemical characteristics and fungal community in the Baishilazi Nature Reserve. *PeerJ* 6:e6251. 10.7717/peerj.6251 30648009 PMC6330947

[B12] Dimitriu P. A. Prescott C. E. Quideau S. A. Grayston S. J. (2010). Impact of reclamation of surface-mined boreal forest soils on microbial community composition and function. *Soil Biol. Biochem.* 42 2289–2297. 10.1016/j.soilbio.2010.09.001

[B13] Edgar R. C. (2013). UPARSE: highly accurate OTU sequences from microbial amplicon reads. *Nat. Methods* 10 996–998. 10.1038/nmeth.2604 23955772

[B14] Egidi E. Delgado-Baquerizo M. Plett J. M. Wang J. Eldridge D. J. Bardgett R. D. (2019). A few *Ascomycota taxa* dominate soil fungal communities worldwide. *Nat. Commun.* 10:2369. 10.1038/s41467-019-10373-z 31147554 PMC6542806

[B15] Elliott D. R. Caporn S. J. M. Nwaishi F. Nilsson R. H. Sen R. (2015). Bacterial and fungal communities in a degraded ombrotrophic peatland undergoing natural and managed re-vegetation. *PLoS One* 10:e0124726. 10.1371/journal.pone.0124726 25969988 PMC4430338

[B16] Goldfarb K. C. Karaoz U. Hanson C. A. Santee C. A. Bradford M. A. Treseder K. K. (2011). Differential growth responses of soil bacterial taxa to carbon substrates of varying chemical recalcitrance. *Front. Microbiol.* 2:94. 10.3389/fmicb.2011.00094 21833332 PMC3153052

[B17] Gong W. Bowa V. M. Zhao C. Cheng Z. Zhang L. (2021). Footwall rock slope stability evaluations at Nchanga open pit mine. Zambia. *Geotech. Geol. Eng.* 39 5753–5765. 10.1007/s10706-021-01864-1862

[B18] Guo C. Zhang F. Wang X. Lu N. (2020). Effects of meteorology and soil on the herb species diversity in plantations in a reclamation area of coal mine after 6 years. *Environ. Sci. Pollut. Res.* 27 24231–24241. 10.1007/s11356-020-08402-8402 32306252

[B19] Hailu Gunnabo A. Geurts R. Wolde-Meskel E. Degefu T. E. Giller K. van Heerwaarden J. (2021). Phylogeographic distribution of rhizobia nodulating common bean (*Phaseolus vulgaris* L.) in Ethiopia. *FEMS Microbiol. Ecol.* 97:fiab046. 10.1093/femsec/fiab046 33724341 PMC8016211

[B20] He B. Cai Y. Ran W. Jiang H. (2014). Spatial and seasonal variations of soil salinity following vegetation restoration in coastal saline land in eastern China. *Catena* 118 147–153. 10.1016/j.catena.2014.02.007

[B21] Hou X. Liu S. Cheng F. Su X. Dong S. Zhao S. (2019). Variability of environmental factors and the effects on vegetation diversity with different restoration years in a large open-pit phosphorite mine. *Ecol. Eng.* 127 245–253. 10.1016/j.ecoleng.2018.12.006

[B22] Hu L. Robert C. A. M. Cadot S. Zhang X. Ye M. Li B. (2018). Root exudate metabolites drive plant-soil feedbacks on growth and defense by shaping the rhizosphere microbiota. *Nat Commun.* 9:2738. 10.1038/s41467-018-05122-5127 PMC6048113 30013066

[B23] Huang Y. Cao Y. Zhou W. Kuang X. Wang F. Bai Z. (2021). Effects of straw biochar on the growth of medicago falcata in the reconstructed soil of grassland mining area. *Acta Ecol. Sin.* 41 588–602. 10.5846/stxb202003150552

[B24] Islam M. S. Haque K. A. Jahan N. Atikullah M. Uddin M. N. Naser A. M. (2022). Soil salinity mitigation by naturally grown halophytes in seawater affected coastal Bangladesh. *Int. J. Environ. Sci. Technol.* 1–10. 10.1007/s13762-022-03912-3917

[B25] James A. H. Paul B. John P. (1996). *Land Restoration and Reclamation: Principles and Practice.* London: Longman Pub Group.

[B26] Lei H. Peng Z. Yigang H. Yang Z. (2016). Vegetation and soil restoration in refuse dumps from open pit coal mines. *Ecol. Eng.* 94 638–646. 10.1016/j.ecoleng.2016.06.108

[B27] Li J. Zheng Y. Yan J. Li H. He J. (2013). Succession of plant and soil microbial communities with restoration of abandoned land in the Loess Plateau. China. *J. Soils Sed.* 13 760–769. 10.1007/s11368-013-0652-z

[B28] Li L. (2017). Effects of NaCl on growth, dry matter and ion accumulation in forage crop *Astragalus adsurgens* seedlings. *DEStech Trans. Comput. Sci. Eng.* 10.12783/dteees/edep2017/15587

[B29] Li P. Zhang X. Hao M. Cui Y. Zhu S. Zhang Y. (2019). Effects of vegetation restoration on soil bacterial communities, enzyme activities, and nutrients of reconstructed soil in a mining area on the Loess Plateau. China. *Sustainability* 11:2295. 10.3390/su11082295

[B30] Li X. Xia J. Zhao X. Chen Y. (2019). Effects of planting Tamarix chinensis on shallow soil water and salt content under different groundwater depths in the Yellow River Delta. *Geoderma* 335 104–111. 10.1016/j.geoderma.2018.08.017

[B31] Liao C. (2009). *Study on Ecological Effects of Mining Landscape and Ecological Restoration in Yangquan Coal Mine Mining Region.* Master’s thesis, Beijing: Tsinghua University.

[B32] Lin Y. Qin F. Zheng Z. Zhang L. Liu L. Xu W. (2015). Characteristics of variations in soil surface micro-topography and soil erosion on the cross ridge slope under different rainfall conditions. *J. Soil Water Conserv.* 13 32–38. 10.3969/j.issn.1672-3007.2015.03.005

[B33] Liu B. (2018). *Ecological Data Analysis and Modeling.* Ningxia: Ningxia People’s Education Press.

[B34] Liu B. Zhao W. Wen Z. Yang Y. Chang X. Yang Q. (2019). Mechanisms and feedbacks for evapotranspiration-induced salt accumulation and precipitation in an arid wetland of China. *J. Hydrol. X* 568 403–415. 10.1016/j.jhydrol.2018.11.004

[B35] Liu J. Sui Y. Yu Z. Shi Y. Chu H. Jin J. (2015). Soil carbon content drives the biogeographical distribution of fungal communities in the black soil zone of northeast China. *Soil Biol. Biochem.* 83 29–39. 10.1016/j.soilbio.2015.01.009

[B36] Liu X. Bai Z. Zhou W. Cao Y. Zhang G. (2017). Changes in soil properties in the soil profile after mining and reclamation in an opencast coal mine on the Loess Plateau. China. *Ecol. Eng.* 98 228–239. 10.1016/j.ecoleng.2016.10.078

[B37] Magoè T. Salzberg S. L. (2011). FLASH: fast length adjustment of short reads to improve genome assemblies. *Bioinformatics* 27 2957–2963. 10.1093/bioinformatics/btr507 21903629 PMC3198573

[B38] Martin D. D. Ciulla R. A. Roberts M. F. (1999). Osmoadaptation in archaea. *Appl Environ. Microbiol.* 65 1815–1825. 10.1128/aem.65.5.1815-1825.1999 10223964 PMC91261

[B39] Massaccesi L. Benucci G. M. N. Gigliotti G. Cocco S. Corti G. Agnelli A. (2015). Rhizosphere effect of three plant species of environment under periglacial conditions (Majella Massif, central Italy). *Soil Biol. Biochem.* 89 184–195. 10.1016/j.soilbio.2015.07.010

[B40] Miller J. Barton C. Agouridis C. Fogel A. Dowdy T. Angel P. (2012). Evaluating soil genesis and reforestation success on a surface coal mine in Appalachia. *Soil Sci. Soc. Am. J.* 76 950–960. 10.2136/sssaj2010.0400

[B41] Neldner V. J. Ngugi M. R. (2017). Establishment of woody species across 26 years of revegetation on a Queensland coal mine. *Ecol. Manag. Restor.* 18 75–78. 10.1111/emr.12243

[B42] Orozco-Aceves M. Tibbett M. Standish R. J. (2017). Correlation between soil development and native plant growth in forest restoration after surface mining. *Ecol. Eng.* 106 209–218. 10.1016/j.ecoleng.2017.06.004

[B43] Peng S. (2000). Restoration ecology and restoration of degraded ecosystem. *Bull. Chin. Acad. Sci.* 3 188–192.

[B44] Qu X. Long H. Xie P. Cao X. Wang J. (2018). Genetic characteristics and classification of typical sierozem in central ningxia, China. *Acta Pedologica Sinica* 55 75–87. 10.11766/trxb201706120097

[B45] Ren C. Wang T. Xu Y. Deng J. Zhao F. Yang G. (2018). Differential soil microbial community responses to the linkage of soil organic carbon fractions with respiration across land-use changes. *For. Ecol. Manag.* 409 170–178. 10.1016/j.foreco.2017.11.011

[B46] Rhoades J. D. Manteghi N. A. Shouse P. J. Alves W. J. (1989). Soil electrical conductivity and soil salinity: new formulations and calibrations. *Soil Sci. Soc. Am. J.* 53 433–439. 10.2136/sssaj1989.03615995005300020020x

[B47] Schulte C. C. M. Borah K. Wheatley R. M. Terpolilli J. J. Saalbach G. Crang N. (2021). Metabolic control of nitrogen fixation in rhizobium-legume symbioses. *Sci. Adv.* 7:eabh2433. 10.1126/sciadv.abh2433 34330708 PMC8324050

[B48] Schulz S. Brankatschk R. Dümig A. Kögel-Knabner I. Schloter M. Zeyer J. (2013). The role of microorganisms at different stages of ecosystem development for soil formation. *Biogeosciences* 10 3983–3996. 10.5194/bg-10-3983-2013

[B49] Shrestha R. K. Lal R. (2011). Changes in physical and chemical properties of soil after surface mining and reclamation. *Geoderma* 161 168–176. 10.1016/j.geoderma.2010.12.015

[B50] Šourková M. Frouz J. Fettweis U. Bens O. Hüttl R. F. Šantručková H. (2005). Soil development and properties of microbial biomass succession in reclaimed post mining sites near Sokolov (Czech Republic) and near Cottbus (Germany). *Geoderma* 129 73–80. 10.1016/j.geoderma.2004.12.032

[B51] Srinivasan M. C. Laxman R. S. Deshpande M. V. (1991). Physiology and nutritional aspects of actinomycetes: an overview. *World J. Microbiol. Biotechnol.* 7 171–184. 10.1007/bf00328987 24424929

[B52] Srivastava D. S. Cadotte M. W. MacDonald A. A. M. Marushia R. G. Mirotchnick N. (2012). Phylogenetic diversity and the functioning of ecosystems. *Ecol. Lett.* 15 637–648. 10.1111/j.1461-0248.2012.01795.x 22583836

[B53] Stackebrandt E. Goebel B. M. (1994). Taxonomic note: a place for DNA-DNA reassociation and 16S rRNA sequence analysis in the present species definition in bacteriology. *Int. J. Syst. Evol. Microbiol.* 44 846–849. 10.1099/00207713-44-4-846

[B54] van der Heijden M. G. A. Bardgett R. D. van Straalen N. M. (2008). The unseen majority: soil microbes as drivers of plant diversity and productivity in terrestrial ecosystems. *Ecol. Lett.* 11 296–310. 10.1111/j.1461-0248.2007.01139.x 18047587

[B55] Verma D. Thareja R. Kainthola A. Singh T. N. (2011). Evaluation of open pit mine slope stability analysis. *Int. J. Earth Sci.* 4 590–600.

[B56] Waldrop M. P. Zak D. R. Blackwood C. B. Curtis C. D. Tilman D. (2006). Resource availability controls fungal diversity across a plant diversity gradient. *Ecol. Lett.* 9 1127–1135. 10.1111/j.1461-0248.2006.00965.x 16972876

[B57] Walker D. J. Clemente R. Bernal M. P. (2004). Contrasting effects of manure and compost on soil pH, heavy metal availability and growth of *Chenopodium album* L. in a soil contaminated by pyritic mine waste. *Chemosphere* 57 215–224. 10.1016/j.chemosphere.2004.05.020 15312738

[B58] Wang B. Liu G. Xue S. (2011). Effect of black locust (*Robinia pseudoacacia*) on soil chemical and microbiological properties in the eroded hilly area of China’s Loess Plateau. *Environ. Earth Sci.* 65 597–607. 10.1007/s12665-011-1107-1108

[B59] Wang W. Zhang D. (2011). Effects of Halogeton arachnoideus on saline soil improvement. *Pratacultural Sci.* 6 902–904.

[B60] Wu G. Wei D. Zhou Z. Tang M. Fu X. (2014). A summary of study on ecological restoration technology of large coal bases construction in China. *Acta Ecol. Sin.* 34 2812–2820. 10.5846/stxb201308092052

[B61] Xian W. Zhang X. Li W. (2020). Research status and prospect on bacterial phylum Chloroflexi. *Acta Microbiol. Sin.* 60 1801–1820. 10.13343/j.cnki.wsxb.20200463

[B62] Xu L. Zhang B. Wang E. Zhu B. Yao M. Li C. (2021). Soil total organic carbon/total nitrogen ratio as a key driver deterministically shapes diazotrophic community assemblages during the succession of biological soil crusts. *Soil Ecol. Lett.* 3 328–341. 10.1007/s42832-020-0075-x

[B63] Yang Y. Liu J. Zhang T. Zheng H. Dong H. Li L. (2017). Effects of different vegetations reclamation on soil physical and chemical properties in Heidaigou opencast coal mine area. *Sustain. Dev.* 07 240–247. 10.12677/sd.2017.74029

[B64] Yao M. Rui J. Li J. Dai Y. Bai Y. Hedìnec P. (2014). Rate-specific responses of prokaryotic diversity and structure to nitrogen deposition in the Leymus chinensis steppe. *Soil Biol. Biochem.* 79 81–90. 10.1016/j.soilbio.2014.09.009

[B65] Yuan Y. Zhao Z. Bai Z. Wang H. Wang Y. Niu S. (2016). Reclamation patterns vary carbon sequestration by trees and soils in an opencast coal mine, China. *Catena* 147 404–410. 10.1016/j.catena.2016.07.039

[B66] Yuan Y. Zhao Z. Niu S. Li X. Wang Y. Bai Z. (2018). Reclamation promotes the succession of the soil and vegetation in opencast coal mine: a case study from *Robinia pseudoacacia* reclaimed forests, Pingshuo mine, China. *Catena* 165 72–79. 10.1016/j.catena.2018.01.025

[B67] Zeng Q. Liu Y. An S. (2017). Impact of litter quantity on the soil bacteria community during the decomposition of *Quercus wutaishanica* litter. *PeerJ* 5:e3777. 10.7717/peerj.3777 28894648 PMC5592084

[B68] Zhalnina K. Louie K. B. Hao Z. Mansoori N. da Rocha U. N. Shi S. (2018). Dynamic root exudate chemistry and microbial substrate preferences drive patterns in rhizosphere microbial community assembly. *Nat. Microbiol.* 3 470–480. 10.1038/s41564-018-0129-123 29556109

[B69] Zhang C. Liu G.-B. Xue S. Xiao L. (2013a). Effect of different vegetation types on the rhizosphere soil microbial community structure in the Loess Plateau of China. *J. Integr. Agric.* 12 2103–2113. 10.1016/s2095-3119(13)60396-60392

[B70] Zhang H. Li G. Song X. Yang D. Li Y. Qiao J. (2013b). Changes in soil microbial functional diversity under different vegetation restoration patterns for Hulunbeier Sandy Land. *Acta Ecol. Sin.* 33 38–44. 10.1016/j.chnaes.2012.12.006

[B71] Zhao S. Liu J.-J. Banerjee S. White J. F. Zhou N. Zhao Z.-Y. (2019). Not by salinity alone: how environmental factors shape fungal communities in saline soils. *Soil Sci. Soc. Am. J.* 83 1387–1398. 10.2136/sssaj2019.03.0082

[B72] Zhao Z. Shahrour I. Bai Z. Fan W. Feng L. Li H. (2013). Soils development in opencast coal mine spoils reclaimed for 1-13 years in the West-Northern Loess Plateau of China. *Eur. J. Soil Biol.* 55 40–46. 10.1016/j.ejsobi.2012.08.006

[B73] Zheng Z. He S. Wu F. (2010). Effects of soil surface roughness on sheet erosion and change under different rainfall conditions. *Trans. Chin. Soc. Agric. Eng.* 26 139–145.

